# Central hyperthyroidism combined with Graves' disease: case series and review of the literature

**DOI:** 10.1530/ETJ-22-0223

**Published:** 2023-07-28

**Authors:** Caiyan Mo, Han Chen, Qi Zhang, Ying Guo, Liyong Zhong

**Affiliations:** 1 Department of Endocrinology, Beijing Tiantan Hospital, Capital Medical University, Beijing, China

**Keywords:** central hyperthyroidism, Graves’ disease, thyroid-stimulating hormone-secreting pituitary adenoma, resistance to thyroid hormone β

## Abstract

**Background:**

Central hyperthyroidism is characterized by elevated free thyroid hormone and unsuppressed thyroid-stimulating hormone (TSH), and this laboratory feature includes TSH-secreting pituitary adenoma (TSHoma) and resistance to thyroid hormone β (RTHβ). Central hyperthyroidism combined with Graves’ disease (GD) has been rarely reported.

**Case Report:**

We describe three patients with TSHoma combined with GD and one patient with GD combined with RTHβ and pituitary adenoma. These three patients with TSHoma combined with GD showed elevated thyroid hormone, while TSH level was normal or elevated, and TSH receptor antibodies were positive. After thyrotoxicosis was controlled, they all underwent transsphenoidal surgery. We also describe a patient with an initial presentation of GD who developed hypothyroidism after anti-hyperthyroidism treatment and TSH was inappropriately significantly increased. His head magnetic resonance imaging revealed a pituitary adenoma. Genetic testing confirmed a heterozygous mutation in the thyroid hormone receptor β gene c.1148G>A (p.R383H). After levothyroxine and desiccated thyroid tablet treatment, the TSH level decreased to normal.

**Conclusion:**

These four cases highlight the need to consider the diagnosis of GD combined with central hyperthyroidism when faced with inconsistent thyroid function test results, illuminating the specific diagnostic and therapeutic challenges of coexisting primary and central hyperthyroidism. Finally, we propose clinical management for central hyperthyroidism combined with GD.

## Introduction

Graves' disease (GD) is an autoimmune disease of the thyroid gland and is the most common cause of hyperthyroidism. The annual incidence is 20–50 cases per 100,000 people, with a peak incidence between the ages of 30 and 50. The binding of thyroid-stimulating hormone receptor antibodies (TRAb) to thyroid-stimulating hormone (TSH) receptors leads to unregulated thyroid hormone production independent of pituitary TSH, resulting in hyperthyroidism (1). Central hyperthyroidism is a rare cause of hyperthyroidism that includes TSH-secreting pituitary adenomas (TSHoma) and resistance to thyroid hormone β (RTHβ). When the thyroid hormone level rises and the TSH concentration is not inhibited, the diagnosis of central hyperthyroidism should be considered.

TSHoma is a rare disease in which pituitary adenomas abnormally overproduce TSH, accounting for 0.5–2% of all pituitary adenomas, with an estimated prevalence of 1–2 cases per 1 million in the general population (2). TSHoma usually presents with signs and symptoms of mild hyperthyroidism, probably related to the reduced biological activity of TSH secreted by the pituitary adenoma, and is often accompanied by goiter (2). Also, TSHoma is often combined with neurological symptoms caused by compression of the pituitary mass (3). The diagnostic criteria include evidence of elevated thyroid hormone concentrations accompanied by inappropriate TSH secretion. Lack of TSH response to thyroid-stimulating hormone releasing hormone (TRH) is also a feature of TSHoma. Most of the reported TSHomas are macroadenomas, and transsphenoidal surgery (TSS) is preferred. However, because macroadenomas usually invade adjacent tissues, the treatment outcome of surgery is often unsatisfactory, with a complete remission rate of only about 40% after tumor resection. If complete remission cannot be achieved with surgical intervention, further radiotherapy or pharmacological treatment such as somatostatin analog (SSA) octreotide, may be considered (4).

RTHβ is a rare autosomal dominant disorder in which abnormalities in the thyroid hormone receptor binding domain result in reduced end-organ responsiveness to thyroid hormones, leading to elevated serum thyroid hormone levels and normal or elevated TSH. The prevalence of RTHβ is estimated to be only 1 in 40,000 people (5). The physiological effects of thyroid hormones are mediated by receptors encoded by different genes: the *THRA* gene, encoding thyroid hormone receptor alpha and the *THRB* gene, encoding thyroid hormone receptor beta subtypes 1 and 2, which regulate gene expression in target tissues (6). RTHβ is primarily caused by germline mutations in *THRB*, which result in a failure of negative feedback regulation of TSH by thyroid hormones and can lead to hypothyroidism (7). Clinical manifestations of RTHβ depend on the severity of the mutation and individual and tissue-specific differences in resistance to thyroid hormones. Elevated thyroid hormone levels compensate for resistance to thyroid hormones, and therefore, RTHβ patients do not have severe hypothyroidism symptoms (8, 9).

Patients with central hyperthyroidism are often misdiagnosed with GD because they may present with goiter, palpitations, and elevated thyroid hormones, which often leads to inappropriate treatment. Therefore, it is important to distinguish between these two disorders. Although GD is a common autoimmune thyroid disorder, the coexistence of the two disorders is extremely rare, complicating diagnosis and treatment and often leading to delayed diagnosis of central hyperthyroidism.

## Case series

### Case 1

**Figure 1:**
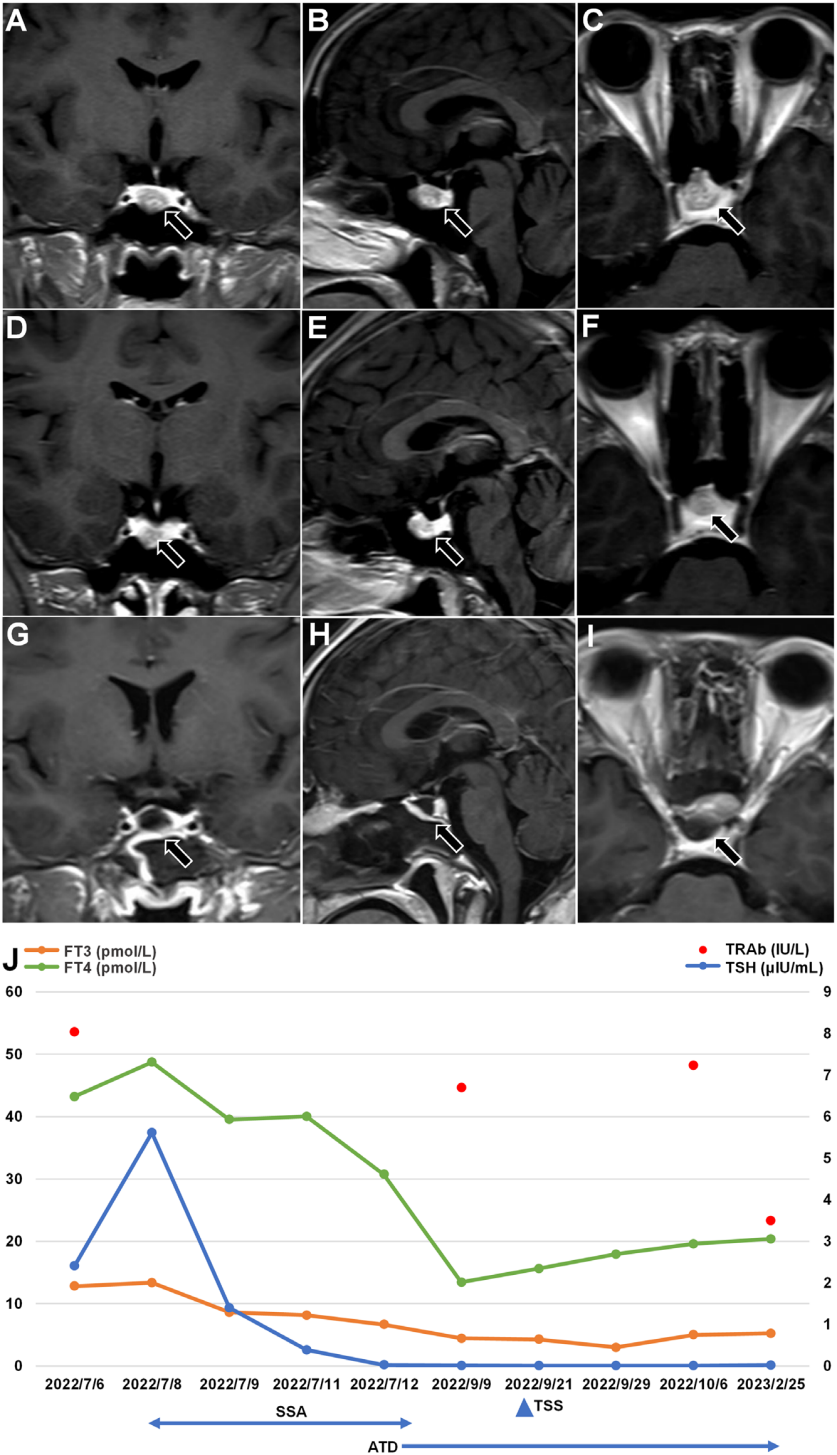
Pituitary enhanced MRI, thyroid function change, and treatment of case 1. ATD, antithyroid drugs; FT3, free tri-iodothyronine; FT4, free thyroxine; SSA, somatostatin analog; TRAb, thyroid-stimulating hormone receptor antibodies; TSH, thyroid-stimulating hormone; TSS, transsphenoidal surgery. Normal ranges: FT4 7.64–16.03 pmol/L, FT3 3.28–6.47 pmol/L, TSH 0.49–4.91 μIU/mL, TRAb 0–1.7 IU/L.

A 26-year-old young woman presented with thyrotoxicosis and headaches from July 2018 and took propranolol irregularly. But the symptoms gradually worsened and developed proptosis, so she came to our department in July 2022, and her thyroid function after admission is shown in Table 1. Unless otherwise specified, the normal ranges of thyroid function are free thyroxine (FT4) 7.64–16.03 pmol/L, free tri-iodothyronine (FT3) 3.28–6.47 pmol/L, total thyroxine (TT4) 69.97–152.52 nmol/L, total tri-iodothyronine (TT3) 1.01–2.48 nmol/L, TSH 0.49–4.91 μIU/mL, thyroglobulin antibody (Tg-Ab) 0–4 IU/mL, thyroid peroxidase antibody (TPO-Ab) 0–9 IU/mL, TRAb 0–1.75 IU/L. The other anterior pituitary hormones, blood count, and liver and kidney function were normal. Ultrasound of the thyroid gland showed a goiter and color Doppler showed abundant blood flow in the gland, which was consistent with the sonographic changes of hyperthyroidism. The pituitary enhanced magnetic resonance imaging (MRI) scan showed a mass-like signal in the sella, approximately 11 × 16 × 17 mm, as shown in Fig. 1A, B and C.

**Table 1:** Clinical manifestations and measurements of patients.

Patient	Age/gender	Clinical manifestations	TT3 (nmol/L)	TT4 (nmol/L)	TSH (μIU/mL)	FT3 (pmol/L)	FT4 (pmol/L)	TRAb (IU/L)	TPO-Ab (IU/mL)	Tg-Ab (IU/mL)	*D*max (mm)	Short-acting octreotide inhibition test
Case 1	26/F	Polyphagia, palpitations, tremor, thermophobia, headache, exophthalmos, and goiter	3.44	239.82	2.407	12.8	43.19	8.04	>1238.00	890.81	17	+
Case 2	47/F	Palpitations, tremor, headache, hypoplasia, and goiter	3.98	201.69	6.854	12.08	30.05	5.06	0.32	1.66	18	+
Case 3	45/F	Palpitations, weight loss, and amenorrhea	4.01	276.86	4.73	14.59	60.03	0.654 (postoperative 3.69)	232.73	14.21	20	+
Case 4	51/M	Palpitations, tremor, and exophthalmos	1.48	96.97	62.635	4.8	14.36	<0.3 (initial positive)	14.44	4.73	12	−

Normal ranges: TT3 1.01–2.48 nmol/L; TT4 69.97–152.52 nmol/L; TSH 0.49–4.91 μIU/mL; FT3 3.28–6.47 pmol/L; FT4 7.64–16.03 pmol/L; TRAb 0–1.7 IU/L; TPO-Ab 0–9 IU/mL; Tg-Ab 0–4 IU/mL.

*D*max, maximum diameter of pituitary adenomas; F, female; FT3, free tri-iodothyronine; FT4, free thyroxine; M, man; Tg-Ab, thyroglobulin antibody; TPO-Ab, thyroid peroxidase antibody; TRAb, thyroid-stimulating hormone receptor antibodies; TSH, thyroid-stimulating hormone; TT3, total tri-iodothyronine; TT4, total thyroxine.

The patient received metoprolol therapy. Since the patient’s T3 and T4 were elevated and TSH was not suppressed, we performed a short-acting octreotide suppression test on day 3 of admission after the patient's informed consent. TSH decreased significantly from 5.613 μIU/mL to 1.8 μIU/mL 2 h after 0.1 mg octreotide subcutaneous injection and to an even lower 0.39 μIU/mL after 72 h. However, FT3 and FT4 decreased insignificantly after octreotide injection and even showed a rebound in FT4 levels after 72 h (Table 2, Fig. 1J). Since TRAb was positive, we considered the combination of GD and therefore the patient was treated with methimazole 20 mg/day on day 7 of admission. The patient did not experience any adverse effects such as nausea, vomiting, or abdominal pain after the short-acting octreotide subcutaneous injection, so she received a long-acting octreotide 20 mg intramuscular injection and continued to take methimazole after discharge.

**Table 2:** Changes of thyroid function before and after short-acting octreotide inhibition test.

Patients	Before SSA			After SSA 72h		
	TSH (μIU/mL)	FT3 (pmol/L)	FT4 (pmol/L)	TSH (μIU/mL)	FT3 (pmol/L)	FT4 (pmol/L)
Case 1	5.613	13.37	48.72	0.39	8.16	40.03
Case 2	6.854	12.08	30.05	0.279	5.72	21.3
	5.077	11.25	27.58	1.555	8.91	25.14
Case 3	4.73	14.59	60.03	1.06	9.26	54.9
Case 4	28.956	4.57	14.73	17.36	3.92	15.2
	6.649	3.96	18.13	7.222	4.35	17.46

Normal ranges: TSH 0.49–4.91 μIU/mL; FT3 3.28–6.47 pmol/L; FT4 7.64–16.03 pmol/L.

FT3, free tri-iodothyronine; FT4, free thyroxine; SSA, somatostatin analog; TSH, thyroid-stimulating hormone.

In September 2022, she returned to our department, and the symptoms of thyrotoxicosis, headache, and proptosis improved. The retested thyroid hormones were normal with suppressed TSH and positive TRAb (Fig. 1J). A repeat pituitary-enhanced MRI scan suggested the lesion size was slightly smaller than before, as shown in Fig. 1D, E and F. TSS was performed in the neurosurgery department of our hospital on September 20, 2022. Postoperative pathology showed pituitary adenoma and immunohistochemistry showed TSH positive. The postoperative pituitary MRI was shown in Fig. 1G, H and I. The postoperative thyroid function was normal, but the TRAb was still positive (Fig. 1J), so she continued to take methimazole. On February 25, 2023, she returned to our department and the thyroid function test showed FT4 20.39 pmol/L, FT3 5.23 pmol/L, TT4 133.13 nmol/L, TT3 1.67 nmol/L, TSH 0.020 μIU/mL, and pituitary MRI showed little change in the lesion from postoperative, indicating remission of TSHoma. Since the retest TRAb was 3.5 IU/L, she continued the treatment with methimazole and was followed up.

### Case 2

**Figure 2:**
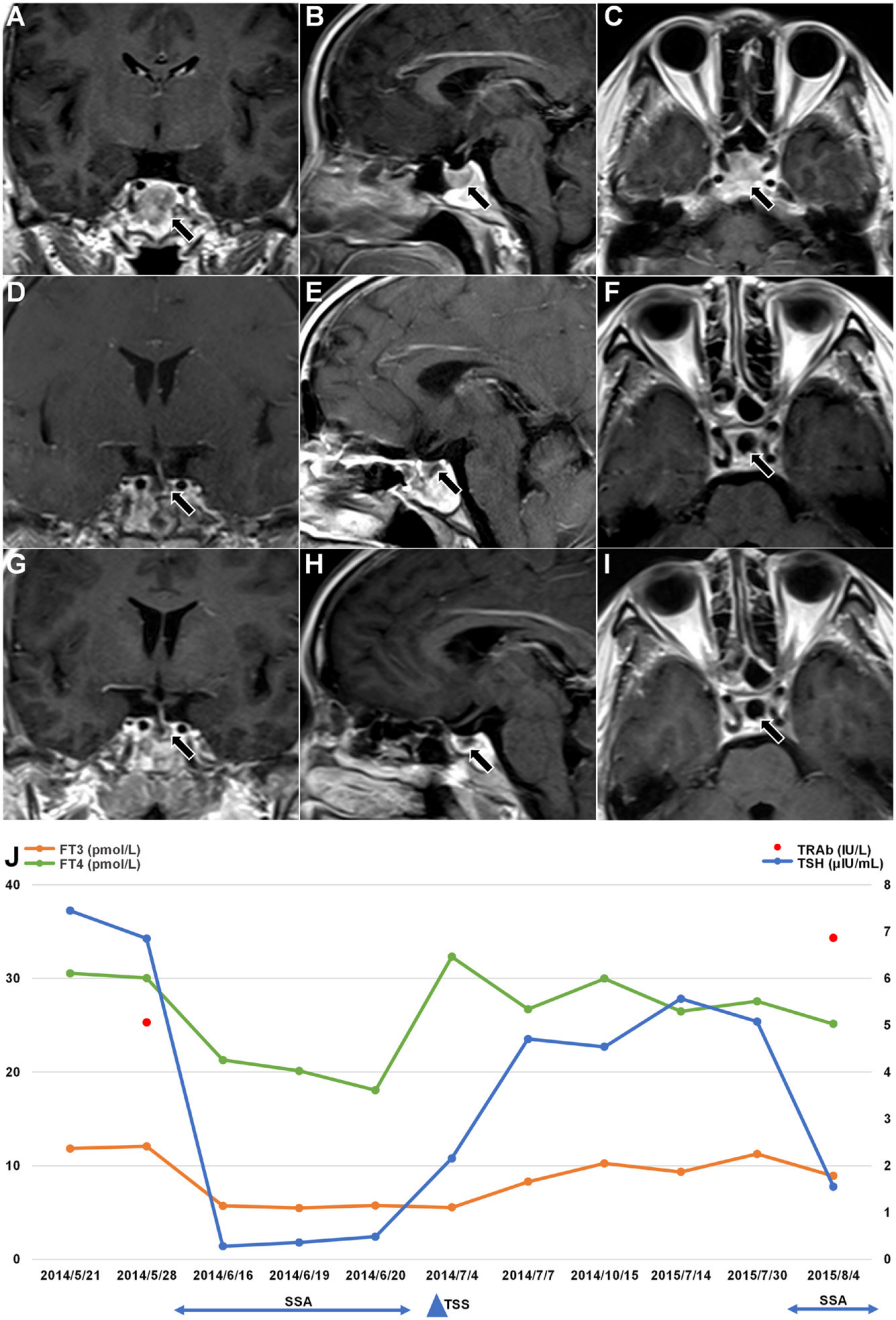
Pituitary enhanced MRI, thyroid function change, and treatment of case 2. FT3, free tri-iodothyronine; FT4, free thyroxine; SSA, somatostatin analog; TRAb, thyroid-stimulating hormone receptor antibodies; TSH, thyroid-stimulating hormone; TSS, transsphenoidal surgery. Normal ranges: FT4 7.64–16.03 pmol/L, FT3 3.28–6.47 pmol/L, TSH 0.49–4.91 μIU/mL, TRAb 0–1.7 IU/L.

A 47-year-old woman developed thyrotoxicosis symptoms with headache and vision loss since May 2014. So she presented to our department, the thyroid function is shown in Table 1, and all other anterior pituitary hormones were normal. Ultrasound of the thyroid gland suggests multiple cystic nodules in the bilateral thyroid gland. Pituitary MRI showed a lump-like signal in the sellar region and sphenoid sinus, about 18 × 18 × 16 mm, and the enhancement scan showed uneven and obvious enhancement, as shown in Fig. 2A, B and C. The patient had elevated T3, T4, and TSH, and the head MRI showed a pituitary tumor, so we considered a possible TSHoma. Therefore, she received 16 days of short-acting octreotide subcutaneous injection and had a significant decrease in thyroid function (Table 2, Fig. 2J). She underwent TSS at our neurosurgery department on July 3, 2014, with postoperative pathology suggesting pituitary adenoma with scattered few positive cells for TSH. Postoperative pituitary MRI is shown in Fig. 2D, E and F, and postoperative thyroid function is shown in Fig. 2J.

However, the patient reported no significant improvement in symptoms after surgery. In July 2015, she returned to our department, and the thyroid function showed FT4 26.49 pmol/L, FT3 9.34 pmol/L, TT4 166.25 nmol/L, TT3 3.59 nmol/L, and TSH 5.57 μIU/mL. The reexamination of the head MRI suggested a recurrence of TSHoma, as shown in Fig. 2G, H and I. Then, we gave her metoprolol to stabilize her heart rate. During her hospitalization, we gave preference to short-acting octreotide to inhibit TSH and observed that she did not have gastrointestinal adverse reactions such as nausea, vomiting, abdominal pain, etc. After 72 h of injection, thyroid function showed FT4 25.14 pmol/L, FT3 8.91 pmol/L, TT4 178.71 nmol/L, TT3 3.00 nmol/L, TSH 1.555 μIU/mL, and TRAb 6.87 IU/L (Table 2, Fig. 2J). Then, we recommended the patient to receive further long-acting octreotide treatment. However, due to the cost, she refused and also declined secondary surgery or gamma-knife treatment, so she continued oral metoprolol after discharge.

### Case 3

**Figure 3:**
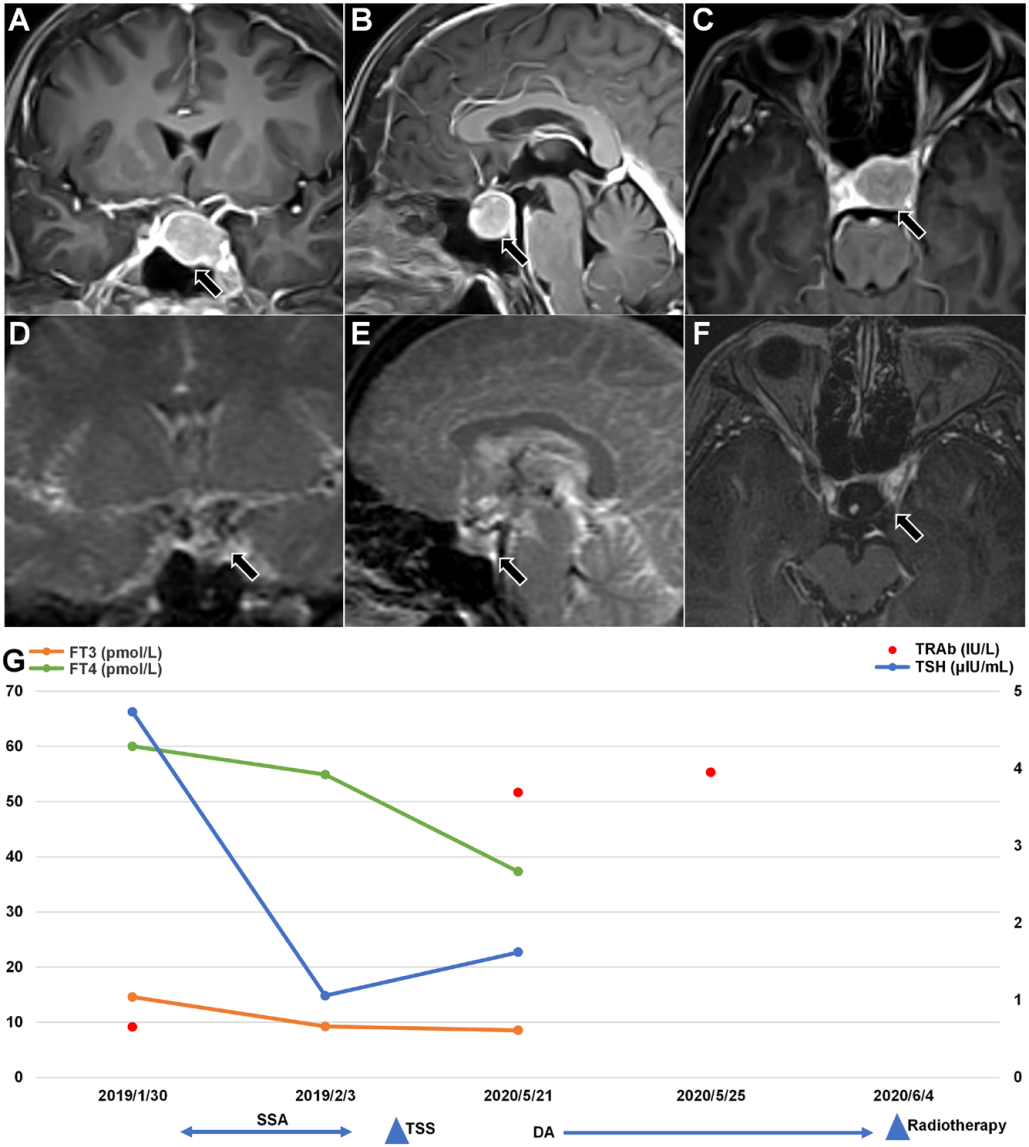
Pituitary-enhanced MRI, thyroid function change, and treatment of case 3. DA, dopamine receptor agonist; FT3, free tri-iodothyronine; FT4, free thyroxine; SSA, somatostatin analog; TRAb, thyroid-stimulating hormone receptor antibodies; TSH, thyroid-stimulating hormone; TSS, transsphenoidal surgery. Normal ranges: FT4 7.64–16.03 pmol/L, FT3 3.28–6.47 pmol/L, TSH 0.49–4.91 μIU/mL, TRAb 0–1.7 IU/L.

A 45-year-old woman had thyrotoxicosis with amenorrhea and was treated with propranolol since November 2018, but the symptoms were not significantly relieved. So she was admitted to our department on January 29, 2019. Thyroid function after admission is shown in Table 1, and the rest of the anterior pituitary hormone levels were normal. Ultrasound of the thyroid gland showed diffuse goiter with multiple cystic nodules (TI-RADS class 2) in the left lobe. Head MRI suggested an oval-shaped adenoma in the sella and supra sella, about 17 × 16 × 20 mm with optic chiasma-compression, as shown in Fig. 3A, B and C. The patient had elevated T3 and T4, TSH was not suppressed, and her head MRI showed a pituitary tumor, we considered the possibility of TSHoma, so short-acting octreotide therapy was given to suppress TSH secretion combined with propranolol to control ventricular rate. No adverse effects were observed and thyroid function was decreased on reexamination (Table 2, Fig. 3G), so she received a deep intramuscular injection of long-acting octreotide before discharge. Then, she underwent TSS at a local hospital on February 19, 2019, with postoperative pathology suggesting TSHoma.

In May 2020, she felt palpitations again and returned to our department, the thyroid function showed FT4 37.32 pmol/L, FT3 8.55 pmol/L, TT4 233.42 nmol/L, TT3 3.05 nmol/L, TSH 1.621 μIU/mL, and TRAb 3.69 IU/L. She had negative TRAb before TSS, but after surgery, her TRAb became positive. A repeat pituitary-enhanced MRI suggested pituitary tumor recurrence. On day 3 after admission, she started metoprolol 25 mg/day and dopamine receptor agonist (DA) bromocriptine titrated gradually from a small dose to 15 mg/day without uncomfortable symptoms such as nausea, headache, or orthostatic hypotension. A retest of TRAb remained positive (3.95 IU/L) on day 6 after admission (Fig. 3G). She underwent gamma-knife treatment at our hospital on June 4, 2020, and the localized head MRI image before radiotherapy is shown in Fig. 3D, E and F. The patient was not subsequently tested for thyroid function and was lost to follow-up.

### Case 4

**Figure 4:**
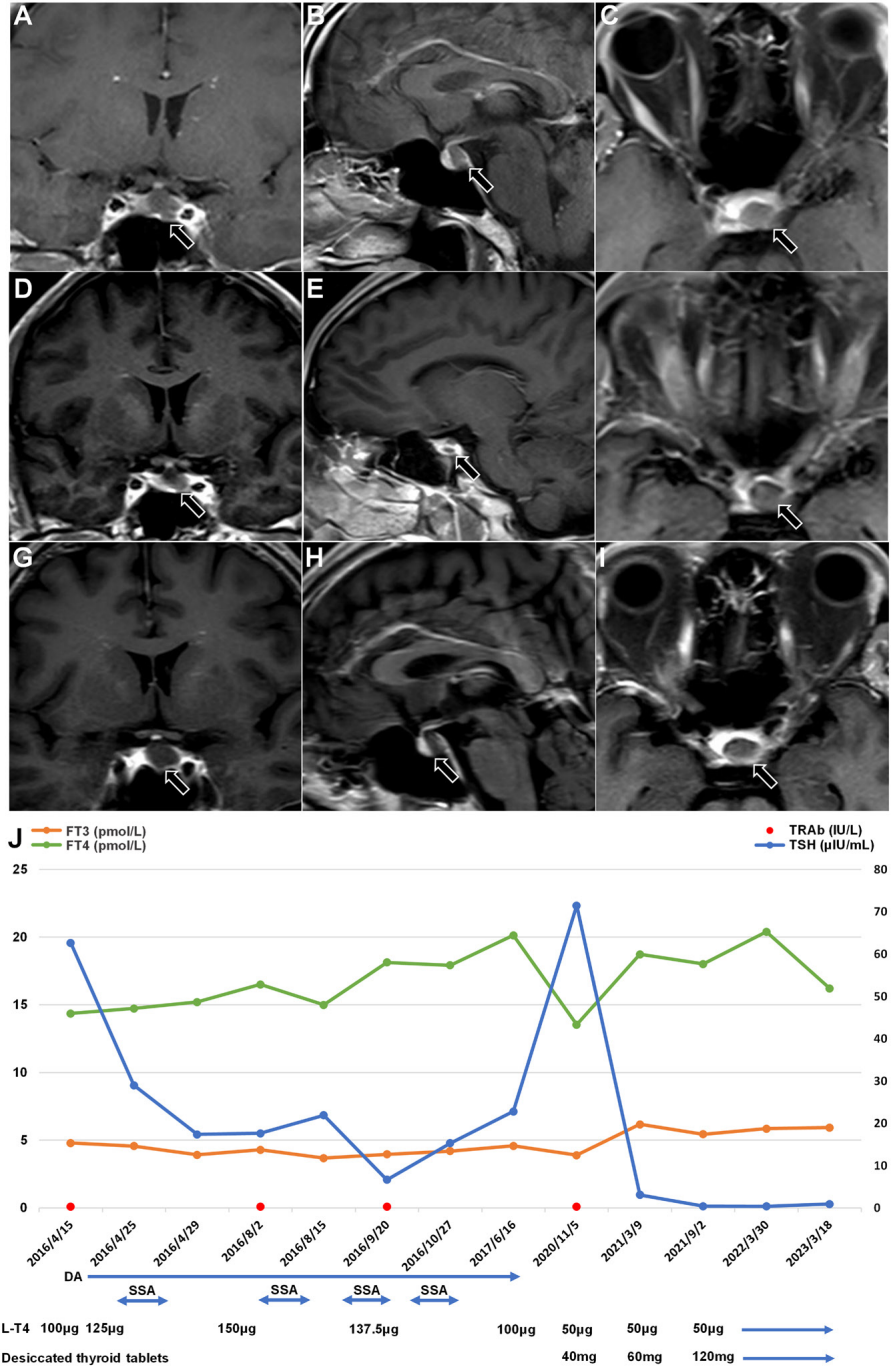
Pituitary-enhanced MRI, thyroid function change, and treatment of case 4. DA, dopamine receptor agonist; FT3, free tri-iodothyronine; FT4, free thyroxine; L-T4, levothyroxine; SSA, somatostatin analog; TRAb, thyroid-stimulating hormone receptor antibodies; TSH, thyroid-stimulating hormone; TSS, transsphenoidal surgery. Normal ranges: FT4 7.64–16.03 pmol/L, FT3 3.28–6.47 pmol/L, TSH 0.49–4.91 μIU/mL, TRAb 0–1.7 IU/L.

A 51-year-old man presenting in another hospital with symptoms of thyrotoxicosis and exophthalmos was diagnosed as having GD. At that time, TSH was suppressed, thyroid hormones were increased, and TRAb was positive. He was intermittently treated with propylthiouracil for 9 years, and lately, he received two doses of radioactive iodine (RAI) in February and November 2009, respectively. Thyroid function was reassessed before RAI with the following results: TT3 11 nmol/L (normal range 0.48–2.01 nmol/L), TT4 279.7 nmol/L (normal range 58.4–124.8 nmol/L), and TSH 0 μIU/mL (normal range 0.35–5.5 μIU/mL). After RAI, refractoriness to levothyroxine (L-T4) replacement treatment was noted, with TSH persistently elevated (16–100 μIU/mL) despite normal or even slightly increased FT4 levels. Since 2014, the patient has had an intermittent headache and decreased vision, the head MRI showed an abnormal signal in the middle of the pituitary, which was not treated. On April 12, 2016, he presented to our department. The thyroid function test is shown in Table 1, and the levels of other anterior pituitary hormones were normal. The thyroid ultrasound showed a small thyroid gland with mild diffuse lesions and rich blood flow signals. Head MRI enhanced scan suggested an abnormal enhancement in the pituitary, about 12 × 8 × 7 mm, considering the possibility of pituitary adenoma, as shown in Fig. 4A, B and C.

The patient took long-term L-T4 100 ug/day replacement therapy before admission. According to the thyroid function, TSH persistently and significantly elevated despite normal or even slightly increased FT4 levels on multiple checks, combined with sinus bradycardia and hyperlipidemia, RTHβ was considered. So L-T4 was increased to 125 ug/day after his admission. Molecular etiology was performed, and a heterozygous mutation in the *THRB* gene c.1148G>A (p.R383H) was detected. First-degree family members of patients with known mutations should also undergo genetic screening to prevent potential misdiagnosis and inappropriate treatment, but case 4 indicated that his family members had normal thyroid function and refused.

Besides, his pituitary MRI indicated a sella region lesion. We initially considered three possibilities: first, pituitary hyperplasia due to RTHβ; second, a proliferation of pituitary TSH cells caused by long-term high levels of TSH secretion, culminating in a tertiary TSHoma; and third, non-functioning pituitary adenoma, which required a series of differential tests. Because of the expression of dopamine 2 receptors on the surface of TSHoma cells, bromocriptine was given from day 4 of admission to inhibit TSH secretion, starting with a small dose and gradually titrated to 15 mg/day, without any adverse reactions such as nausea and headache. After the 12th day of admission, TSH decreased from 62.635 μIU/mL to 28.956 μIU/mL. Because of the somatostatin receptors (SSTRs) expression on the surface of TSHoma cells, the patient began to receive short-acting octreotide subcutaneous injections on the 13th day of admission. After 3 days, the thyroid function showed that TSH further decreased to 17.36 μIU/mL (Table 2, Fig. 4J). The patient was advised to undergo surgery to further clarify the pathological type, but he declined.

He was treated monthly with octreotide in our department from August to October 2016, during which the short-acting octreotide suppression test was performed again, and the results suggested that TSH was not significantly suppressed (Table 2). The dosage of L-T4 was titrated according to his thyroid function, while he continued to take bromocriptine 15 mg/day. A head MRI in August 2016 showed a lesion size of approximately 10 × 10 × 11 mm, as shown in Fig. 4D, E and F. After October 2019, the patient stopped bromocriptine and octreotide on his own and only took L-T4 100 ug/day. He returned to our department again in November 2020, with a repeat thyroid function suggesting FT4 13.53 pmol/L, FT3 3.89 pmol/L, TT4 126.55 nmol/L, TT3 1.38 nmol/L, TSH 71.466 μIU/mL, and TRAb <0.3 IU/L (as in Fig. 4J). A repeat head MRI suggested that the lesion size was about 11 × 8 × 4 mm, slightly smaller than before, as shown in Fig. 4G, H and I. Despite long-term treatment with a supra-physiological dose of L-T4, the patient still had persistent high TSH levels. Since TSH levels in patients with TSHoma are mostly normal or slightly elevated, but the TSH levels of case 4 were far higher than the upper limit of the normal range and could not be adequately suppressed by twice short-acting octreotide inhibition tests, we ruled out the possibility of TSHoma. The assay indicated that the FT4 level was higher than the normal range, while the FT3 level was near the low limit of the normal range. Since about 80% of FT3 is converted from FT4 in the periphery; therefore, he was then started on L-T4 50 ug/day combined with 40 mg/day desiccated thyroid tablets gradually titrated to 60 mg/day to suppress TSH. After discharge, we conducted a long-term follow-up and continued to titrate the dosage of L-T4 and desiccated thyroid tablets according to his thyroid function. The patient is currently taking L-T4 50 ug/day and desiccated thyroxine tablets 120 mg/day and the thyroid function showed that FT3 5.94 pmol/L (2.43–6.01 pmol/L), FT4 16.20 pmol/L (9.01–19.05 pmol/L), and TSH 0.916 μIU/mL (0.35–4.94 μIU/mL), and the head MRI showed no change in the size of the lesion. Therefore, we finally considered that the sellar region lesions of this patient included two situations. After we successively used L-T4 and desiccated thyroid tablets to inhibit the TSH level, the size of sellar region lesions was slightly smaller than before, indicating that some lesions originate from pituitary hyperplasia caused by RTHβ. When the TSH level dropped to the normal range, it was observed that the size of the sella region lesion did not change after a long-term follow-up, and the patient had no evidence of other anterior pituitary hormone hypersecretion. So the residual sella region lesion was considered more likely a non-functioning pituitary adenoma. Unfortunately, because the patient refused to operate, we could not get the pathological results to further confirm our diagnosis.

### Review of the literature

We searched PubMed for cases of TSHoma combined with GD. Only 16 cases have been reported so far, as shown in Table 3. Among them, TSHoma preceded the development of GD in five cases. GD occurred before the onset of TSHoma in five cases. In the other six cases, GD and TSH tumors may have coexisted at the time of initial diagnosis. The relationship between TSHoma and GD is controversial. It is unclear whether TSHoma and GD coexist by chance or whether there is some mechanism linking TSHoma and GD. More evidence is needed to confirm the association between GD and TSHoma.

**Table 3:** Case reports of TSHoma combined with GD.

	Authors	Year	Country	Age/gender	Clinical process	Clinical manifestations	TSH (μIU/mL)	*D*max (mm)	Treatment measures	Management and follow-up
1	O’Donnel *et al*. (10)	1973	UK	25/M	TSHoma→GD	Thyrotoxicosis signs, scant hair growth, impairment of peripheral vision	28	N/A	Cortisol, testosterone, ATD, thyroxine, hypophysectomy	Recurrent hyperthyroidism, recovered after ATD treatment
2	Sandler *et al*. (11)	1976	USA	56/F	GD complicated with TSHoma	Thyrotoxicosis signs, symptomatic ophthalmopathy, and acromegaly	8.1	N/A	ATD, pituitary irradiation, propranolol, radioiodine therapy, cortisone	Recurrent hyperthyroidism several times, intermittent ATD treatment, TSH levels at or below detectable limits
3	Yovos *et al*. (12)	1981	USA	17/F	GD complicated with TSHoma	Thyrotoxicosis signs, unilateral exophthalmos, and goiter	52.3	N/A	ATD, propranolol, iodides, steroids, craniotomy, cranial and orbital irradiation	Hyperthyroidism improved, taking only propranolol
4	Kamoi *et al*. (13)	1985	Japan	46/F	TSHoma→GD	Thyrotoxicosis signs, goiter, and galactorrhoea	15.5	N/A	ATD, prednisolone, TSS	Discontinuation of ATD-induced recurrent hyperthyroidism without evidence of regrowth of the tumor
5	Koriyama *et al*. (14)	2004	Japan	31/F	TSHoma→GD	Thyrotoxicosis signs and goiter	2.1	N/A	Octreotide and L-T4, ATD, TSS (twice)	Discontinue treatment due to financial reasons and the absence of severe thyrotoxic symptoms
6	Kageyama *et al*. (15)	2007	Japan	21/F	TSHoma→GD	Thyrotoxicosis signs	3.16	10	TSS	Recurrent hyperthyroidism
7	Lee & Wang (16)	2010	China	27/M	GD→TSHoma	Thyrotoxicosis signs and goiter	<0.004	10.4	ATD	Recurrent hyperthyroidism, refused surgery and continued ATD treatment
8	Lee & Wang (16)	2010	China	28/F	GD→TSHoma	Thyrotoxicosis signs and goiter	0.123	15	ATD	TSH was normal, but FT4 was still high, refused surgery
9	Ogawa & Tominaga (17)	2013	Japan	32/F	GD→TSHoma	Thyrotoxicosis signs	Less than detectable	5	ATD, TSS	Recovery
10	Kamoun *et al*. (18)	2013	France	36/F	GD→TSHoma	Thyrotoxicosis signs, goiter, and exophthalmos	1.2–1.8	10	ATD, propranolol, thyroid lobectomy, lanreotide, TSS	Recovery
11	Okuyucu *et al*. (19)	2016	Turkey	37/F (pregnant)	GD complicated with TSHoma	Thyrotoxicosis signs, goiter, and exophthalmos	5.54	13	ATD, thyroidectomy, TSS	N/A
12	Arai *et al*. (20)	2017	Japan	40/F	GD complicated with TSHoma	Headache and exophthalmos	0.27	13	ATD, TSS	Recovery
13	Li *et al*. (21)	2018	China	55/M	GD complicated with TSHoma	Recurrent atrial fibrillation and thyrotoxicosis signs	8.9	23	ATD, TSS	Tumor recurrence and thyroid function tests remained clinically acceptable, remained ATD treatment
14	Campi *et al*. (22)	2020	Italy	36/N/A	interf→TSHoma→GD	Thyrotoxicosis signs	1.290	N/A	SMS-LAR, ATD	Recurrent hyperthyroidism after ATD was discontinued, while TSH levels were normal
15	Fu *et al*. (23)	2020	China	55/F	GD complicated with TSHoma	Thyrotoxicosis signs and goiter	0.337	17	Propranolol, TSS, ATD (postoperative)	TSH below normal range, continued ATD therapy
16	Quinn *et al*. (24)	2020	Ireland	68/F	GD→TSHoma	asymptomatic	<0.02	N/A	ATD, beta-adrenergic blocker	Declined pituitary surgery, managed with a beta-adrenergic blocker and was clinically euthyroid
17	Case 1	2022	China	26/F	GD complicated with TSHoma	Thyrotoxicosis signs, headache, exophthalmos, and goiter	2.407	17	Propranolol, metoprolol, octreotide, ATD, TSS	Thyroid function was normal, TRAb was still positive, continued ATD treatment
18	Case 2	2022	China	47/F	GD complicated with TSHoma	Thyrotoxicosis signs, headache, hypopsia, and goiter	6.854	18	Metoprolol, octreotide, TSS	Recurrence of hyperthyroidism and tumor, refusal of SMS-LAR, secondary surgery, or radiation therapy for financial reasons, continued metoprolol treatment
19	Case 3	2022	China	45/F	TSHoma→GD	Thyrotoxicosis signs and amenorrhea	4.73	20	Propranolol, octreotide, TSS, bromocriptine, metoprolol, gamma-knife	Recurrence of hyperthyroidism and tumor, treated with metoprolol, bromocriptine, and gamma-knife

ATD, antithyroid drugs; *D*max, maximum diameter of pituitary adenomas; F, female; FT4, free thyroxine; GD, Graves' disease; L-T4, levothyroxine; M, man; N/A, not available; SMS-LAR, long-acting-release somatostatin analog; TRAb, thyroid-stimulating hormone receptor antibodies; TSH, thyroid-stimulating hormone; TSHoma, thyroid-stimulating hormone-secreting pituitary adenoma; TSS, transsphenoidal surgery.

We also searched PubMed for cases of RTHβ combined with GD. Only eight cases have been reported so far. Of these, GD preceded RTHβ in six cases, and RTHβ was diagnosed earlier or simultaneously with GD in two cases, as shown in Table 4.

**Table 4:** Case reports of RTHβ combined with GD.

	Authors	Year	Country	Age/gender	Clinical manifestations	Clinical course	TSH variations (μIU/mL)	Family history	*THRB* mutation	Treatment	Management and follow-up
1	Pillay *et al*. (25)	1986	South Africa	24/F (pregnant)	Signs of thyrotoxicosis, goiter, and exophthalmos	GD→RTHβ	N/A	Brother	N/A	Propranolol, ATD	Symptomatic control but biochemically remained hyperthyroid
2	Sato (26)	2010	Japan	34/F	Signs of thyrotoxicosis and goiter	RTHβ+ Hashimoto’s thyroiditis→GD	1.124→1.795	Father	P453T	ATD	Two years after cessation of ATD, GD remission
3	Sivakumar & Chaidarun (27)	2010	Lebanon	53/M	Signs of thyrotoxicosis and goiter	GD→RTHβ	0.2→31.5	No	G347W	^131^I ablation of the thyroid gland, L-T4	Recovery
4	Ogawa *et al*. (28)	2011	Japan	17/M	Signs of thyrotoxicosis and goiter	GD→RTHβ	<0.05→8.308	No	A234T	ATD	Thyroid function almost within the appropriate range, and GD near remission
5	Shiwa *et al*. (29)	2011	Japan	33/F	Signs of thyrotoxicosis and goiter	GD→RTHβ	0.007→107.7	N/A	G251R	ATD	GD remission
6	Ramos-Leví *et al*. (30)	2016	Spain	46/F	Signs of thyrotoxicosis, goiter, headache, and atrial fibrillation	RTHβ complicated with pituitary adenoma and GD	2.86→20	N/A	p.P453R	Cabergoline, ATD, ^131^I therapy, L-T4	Thyroid hormone still elevated but remained asymptomatic
7	Sun *et al*. (31)	2017	China	14/F	Signs of thyrotoxicosis and goiter	GD→RTHβ	0.037→75.52	Father	A *THRB* mutation in Exon 10	ATD, propranolol	Asymptomatic with slightly elevated FT3 and FT4 levels, and a normal TSH level
8	Akahori & Usuda (7)	2021	Japan	30/M	Signs of thyrotoxicosis and goiter	GD→RTHβ	<0.35→4.77	No	R282S	ATD	Thyroid hormone fluctuated after cessation of ATD but no signs of thyrotoxic symptoms
9	Case 4	2022	China	51/M	Signs of thyrotoxicosis and exophthalmos	GD→RTHβ	Less than detectable→>150	N/A	p.R383H	ATD, ^131^I therapy, L-T4, bromocriptine, octreotide, and desiccated thyroxine tablets	Recovery

ATD, antithyroid drugs; FT3, free tri-iodothyronine; FT4, free thyroxine; GD, Graves' disease; L-T4, levothyroxine; N/A, not available; RTHβ, resistance to thyroid hormone β; *THRB*, thyroid hormone receptor β; TRAb, thyroid-stimulating hormone receptor antibodies; TRH, thyroid-stimulating hormone releasing hormone; TSH, thyroid-stimulating hormone.

## Discussion

### Pathogenesis

TSH plays an important role in maintaining normal physiology and regulating the expression of immunoregulatory genes in thyroid cells. In cases where TSHoma precedes the development of GD, investigators have suggested that either sustained elevation or marked fluctuations in TSH may induce GD. Abnormal hypersecretion of TSH can produce anti-specific antibodies that lead to GD (13). Normalizing TSH secretion by treating TSHoma may reduce the production of these antibodies and thus ameliorate Graves’ hyperthyroidism. A sharp postoperative decrease in TSH levels may also induce GD. Some reports suggest that GD occurs after surgical treatment of TSHoma, as thyroid autoantibodies tend to be elevated after pituitary surgery (11). Fas antigen is functionally expressed on the surface of thyroid cells, and TSH inhibits Fas antigen-mediated apoptosis of thyroid cells and promotes thyroid growth (32). Intercellular adhesion molecule-1 (ICAM-1) and major histocompatibility complex (MHC) II molecules are thought to be causative factors in autoimmune thyroid disease (AITD), and their expression can be suppressed by TSH (33). Thus, a rapid decrease in TSH levels after TSS, pituitary irradiation, or octreotide application may induce apoptosis and activate autoimmune responses against the thyroid by increasing the expression of Fas as well as ICAM-1 and MHC II molecules on the thyroid cell surface (14). Physicians should pay attention to changes in thyroid-related autoantibodies after treatment of TSHoma, especially the development of GD. Besides, TSHomas were diagnosed in some cases after the diagnosis of GD. Some investigators believe that the initial treatment with ATD for GD may actually promote the growth of subsequent TSHoma due to a positive feedback system (19).

A high prevalence of TPO-Ab and Tg-Ab has been reported in RTHβ patients, suggesting a potential interaction between RTHβ and AITD (34). Recently, it has been shown that chronic TSH stimulation in RTHβ activates intrathyroidal lymphocytes, leading to thyroid injury and AITD (35). Moreover, it has been shown that thyroid hormone activates the immune system by acting on thymic epithelial cells and directly on neutrophils, natural killer cells, macrophages, and dendritic cells (36). Thyroid hormones enhance dendritic cell maturation and induce pro-inflammatory and cytotoxic responses. Given the involvement of dendritic cells in the pathogenesis of AITD (37), this may also be the pathway that mediates the association between RTHβ and AITD. However, further clinical data accumulation is needed to determine whether *THRB* germline mutations lead to AITD.

### Differential diagnosis

Potential interferences in thyroid function tests, including macro-TSH or human anti-mouse IgG antibody, should be suspected when clinical or biochemical differences occur (38). Therefore, we recommend serial dilution and measuring thyroid function with different analytical platforms to exclude assay interference. Besides, it has been reported that elevated serum iodothyronine levels in the absence of thyrotoxicosis may exist, called familial dysalbuminemic hyperthyroxinemia (FDH) due to variants in the gene encoding albumin (*ALB*) (39). Given the higher prevalence of FDH in the general population with respect to RTHβ, itself more prevalent than TSHoma, we recommend to then search for an *ALB* variant and sequence the *THRB* gene, which will avert submitting many patients to unnecessary and costly MRI and avoid misdiagnoses and/or mistreatments (40).

It is important to distinguish between primary and central hyperthyroidism because they are treated in distinctly different ways. The coexistence of TSHoma with primary thyroid diseases such as autoimmune hypothyroidism (41), and GD (18) has been previously reported in the literature and complicates diagnosis and treatment. When the level of thyroid hormone is elevated and TSH is normal or elevated, central hyperthyroidism should be considered. When combined with positive TRAb, coexisting primary hyperthyroidism should be considered. At this time, TSH levels can be suppressed, normal, or slightly elevated (23). After the diagnosis of central hyperthyroidism, further differentiation between TSHoma and RTHβ is required. Computed tomography or MRI can identify most radiological evidence of pituitary tumors, although the interpretation of a pituitary mass can be difficult in some cases (22). High carboxy-terminal cross-linked telopeptide of type I collagen, sex hormone binding globulin, α subunit levels, and high α subunit/TSH ratios can also be used for the auxiliary diagnosis of TSHoma (4, 42). TSHoma is characterized by the expression of SSTRs, especially SSTR2 and SSTR5. SSA is commonly used in the differential diagnosis of central hyperthyroidism and the treatment of TSHoma (43). TSH levels in patients with TSHoma can be suppressed by short-acting octreotide and do not respond to T3 inhibition tests or TRH stimulation tests, but it should be noted that T3 inhibition tests are contraindicated in the elderly and in patients at high cardiovascular risk. Among the previously reported cases of TSHoma combined with GD, six cases (11, 12, 13, 14, 15, 22) underwent TRH stimulation tests and three of them (13, 15, 22) also underwent T3 inhibition tests. However, TRH is not available in some countries, and T3 inhibition tests are valuable in these cases. In our country, both TRH and T3 were not available, but in case 4, we successfully suppressed TSH by using desiccated thyroid tablets thus indirectly corroborating the diagnosis of RTHβ. Short-acting octreotide inhibition tests were performed in cases 1, 2, 3, and in four previously reported cases (14, 15, 18, 23), but the test also had some limitations. It seems that the SSA test performed poorly in TSHomas with mixed hormone secretion, possibly due to the high expression of dopamine receptors and low expression of SSTRs (44, 45). A decrease in TSH after short-acting SSA trials has also been observed in some RTHβ patients. In Han’s study (46), both TSHoma and RTHβ patients showed a decrease in TSH at the beginning of the SSA trial, but the rate of TSH inhibition in RTHβ patients decreased slowly at 2 h after SSA trials, with TSHoma at 2 and 0 h TSH inhibition rates of 70.58 ± 18.6% and 79.83 ± 12.79%, which were significantly higher than those of 6.01 ± 25.41% and 51.16 ± 13.62% in the RTHβ group (*P* < 0.0001). It suggested that the decrease in TSH levels in RTHβ patients in the short-acting octreotide trial was insignificant, unsustainable, and unstable. Therefore, long-acting SSA is sometimes used as a substitute for short-acting octreotide inhibition tests. For example, in Campi’s study (22), taking long-acting SSA for 2 consecutive months made the correct diagnosis of the patients with TSHoma, which was invisible on MRI. Mannavola (43) found a significant reduction in FT3 and FT4 after two injections of long-acting octreotide in patients with TSHoma, but not in patients with RTHβ, suggesting that administration of long-acting SSA for at least 2 months can differentiate between TSHoma and RTHβ. Besides, RTHβ is usually inherited in an autosomal dominant manner and about 90% of RTHβ patients have a *THRB* mutation (47), so a family history and *THRB* gene testing can help in differential diagnosis.

## Treatment

No clear treatment strategy was found for the coexistence of TSHoma and GD. For patients with TSHoma, TSS is usually the first choice, but to prevent intraoperative and postoperative hyperthyroid crisis, medication needs to be applied to normalize thyroid function before surgery. SSA has been proven to inhibit the secretion of TSH, alleviate hyperthyroidism symptoms, and reduce the size of TSHoma. Therefore, SSA was preferred for preoperative treatment to control thyrotoxicosis in cases 1, 2, 3, and in some of the previously reported cases (14, 18, 22). In other reported cases (17, 21), the ATD first selected for GD failed to normalize the levels of FT4, FT3, and TSH, but led to the progression of TSHoma by inhibiting negative feedback. However, in case 1, two points were unusual. First, she had a positive TRAb with mild hyperthyroidism proptosis, providing clear evidence for GD. Second, in the octreotide suppression test, although TSH decreased significantly to less than 50% of the baseline, FT4 decreased insignificantly and even showed a rebound later in the test, which was considered to be caused by concomitant GD. Therefore, we added methimazole and β-blockers to the application of SSA to counteract the symptoms of thyrotoxicosis. Eventually, her thyroid function was normalized preoperatively and the tumor volume was reduced, and then the TSHoma was successfully removed by endoscopic TSS. However, in cases 2 and 3, despite the positive TRAb, their FT3, FT4, and TSH decreased significantly with octreotide. Therefore, only octreotide was used instead of ATD.

Only a few reports of GD complicated by RTHβ have been reported, and the optimal treatment remains unknown. In case 4 and previously reported cases with both GD and RTHβ, ATD was selected as the initial treatment for GD, but there are no uniform criteria based on clinical symptoms and thyroid function, and radioiodine therapy and thyroidectomy are not recommended. Besides, ATD treatment failed to normalize FT3, FT4, and TSH levels due to the failure of the negative feedback regulatory system of RTHβ (25, 30, 31). Patients with RTHβ usually have elevated FT3 and FT4 levels to compensate for resistance to thyroid hormones. In case 4, pre-admission ATD and RAI treatment resulted in remission of GD, and later hypothyroidism developed and he received L-T4 and T3 replacement therapy. Therefore, the dose of ATD should be controlled during the hyperthyroid phase to make TRAb negative and avoid drug overdose. The dose of L-T4 and T3 should also be titrated promptly if hypothyroidism develops to make the FT3 and FT4 levels close to the upper limit of the normal range or slightly elevated and normalize TSH levels. TSH levels and symptomatic improvement may be important factors in assessing GD remission.

## Clinical management and long-term follow-up

We believe that thyroid function test results should be thoroughly evaluated in the diagnosis of hyperthyroidism and need to be alert to the coexistence of GD and central hyperthyroidism, although this condition is extremely rare. It is the coexistence of these two disorders that complicates diagnosis and treatment and, as a result, thyrotoxicosis has not been adequately controlled in some reports (17, 21). Careful follow-up during antihyperthyroidism therapy is necessary to determine whether TSH secretion is inappropriately regulating thyroid hormones. Moreover, patients who occasionally present with central hyperthyroidism should be considered for coexisting GD. Thus, we propose clinical management for GD combined with central hyperthyroidism.

When a patient’s thyroid function suggests elevated FT3 and FT4 with positive TRAb, first note whether TSH is within the normal range. When TSH is not suppressed, the possibility of central hyperthyroidism should be considered after excluding assay interference and a series of differential tests should be performed. When TSH is significantly suppressed by octreotide accompanied by a significant decrease in FT4, it indicates that the effect of thyroid autoantibodies is less, so surgery is preferred after the application of octreotide to control thyrotoxicosis. When FT4 level does not decrease along with the decrease of TSH after octreotide injection, or even increases instead, suggesting a greater effect of autoantibodies. In this situation, under the close monitoring of thyroid function and imaging, combined treatment of ATD can be given on the basis of SSA to suppress thyrotoxicosis, normalize thyroid function, reduce tumor volume and the risk of anesthesia for further safe resection of tumor by TSS. After successful removal of the tumor, dynamic monitoring of thyroid function and TRAb is still required. Due to the use of ATD, adverse drug reactions, such as blood routine and liver function, also need to be monitored. When FT4 and TSH levels are near the low limit of the normal range, TRAb turns negative, and the review of pituitary MRI shows no obvious residual or recurrent signs of the tumor, considering that both TSHoma and GD are in remission (1, 2). If postoperative central hyperthyroidism persists, SSA combined with ATD therapy may be continued, and changes in the above indicators monitored.

**Figure 5:**
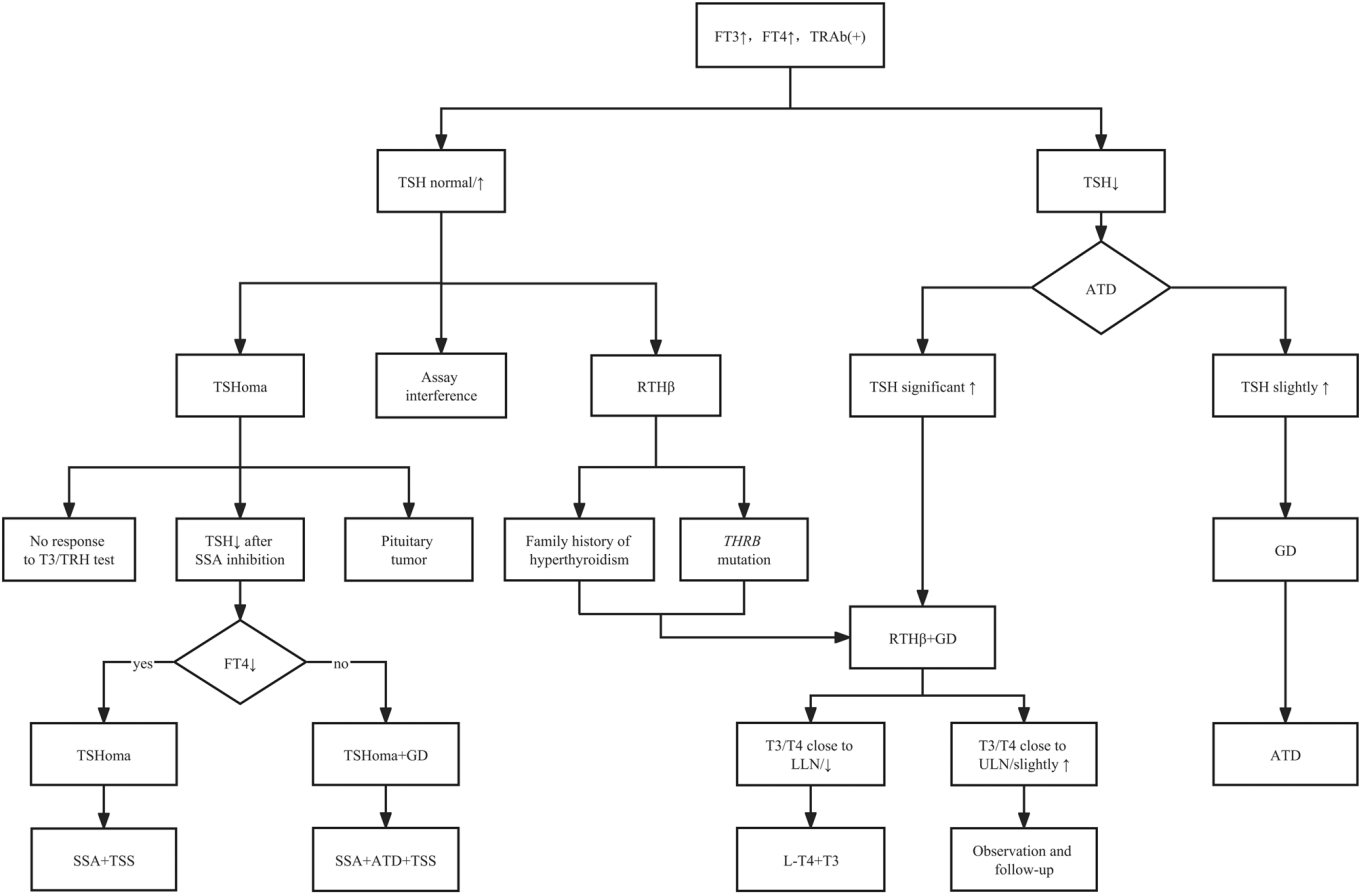
Clinical management plan for GD combined with central hyperthyroidism. ATD, antithyroid drugs; FT3, free tri-iodothyronine; FT4, free thyroxine; GD, Graves' disease; LLN, low limit of the normal range; L-T4, levothyroxine; RTHβ, resistance to thyroid hormone β; SSA, somatostatin analog; T3, tri-iodothyronine; *THRB*, thyroid hormone receptor β; TRAb, thyroid-stimulating hormone receptor antibodies; TRH, thyroid-stimulating hormone releasing hormone; TSH, thyroid-stimulating hormone; TSHoma, pituitary TSH adenoma; TSS, transsphenoidal surgery; ULN, upper limit of normal range.

On the other hand, when FT3 and FT4 are elevated and TRAb is positive with suppressed TSH, in most cases we first consider GD and treat with ATD, which is often accompanied by a mild increase in TSH during the decline in T3 and T4. When RTHβ is combined with GD, it also usually presents with elevated thyroid hormone levels and suppressed TSH levels before treatment, resulting in a delay in the correct diagnosis of RTHβ. When thyroid hormone levels begin to decline or return to normal with antihyperthyroid therapy, inappropriately significant elevations in TSH levels eventually allow for a definitive diagnosis of RTHβ. Therefore, although rare, careful follow-up after initial treatment for GD is necessary. Furthermore, because RTHβ patients are sometimes misdiagnosed with GD, many of them undergo radioiodine ablation of the thyroid or thyroidectomy with a subsequent need for thyroid hormone supplementation. Patients who require supplementation at doses much higher than the estimated dose based on calculated body weight to maintain normal clinical thyroid function, or who continue to have elevated TSH levels despite high-dose supplementation, should also undergo octreotide suppression testing and RTHβ genetic testing. And the treatment plan should bring FT3 and FT4 levels close to the upper limit of the normal range or slightly elevated, normalize TSH levels, turn TRAb negative, avoid ATD overdose, and supplement with L-T4 and T3 if necessary (as shown in Fig. 5).

## Conclusion

We report four rare cases of central hyperthyroidism combined with GD. It is suggested that when faced with inconsistent thyroid function test results, the possibility of central hyperthyroidism combined with primary hyperthyroidism should be noted, as it may confuse the treatment course of hyperthyroidism and complicate the diagnosis and treatment. Thus, we propose clinical management for the combination of GD with central hyperthyroidism, which will help clinicians to identify the possibility of the coexistence of these two disorders on time and to further treat them more appropriately.

## Declaration of interest

The authors declare that there is no conflict of interest that could be perceived as prejudicing the impartiality of this review.

## Funding

This work did not receive any specific grant from any funding agency in the public, commercial, or not-for-profit sector.

## Statement of ethics

The study was approved by the Human Research Ethics Committee of Beijing Tiantan Hospital, Capital Medical University. Written informed consent was obtained from all participants in the study.
